# Health care reform and job satisfaction of primary health care physicians in Lithuania

**DOI:** 10.1186/1471-2296-6-10

**Published:** 2005-03-07

**Authors:** Ilona Buciuniene, Aurelija Blazeviciene, Egle Bliudziute

**Affiliations:** 1 International School of Management, Kaunas, Lithuania; 2 Philosophy and Social Science Department, Kaunas Medical University, Kaunas, Lithuania; 3 Social Medicine Department, Kaunas Medical University, Kaunas, Lithuania

## Abstract

**Background:**

The aim of this research paper is to study job satisfaction of physicians and general practitioners at primary health care institutions during the health care reform in Lithuania.

**Methods:**

Self-administrated anonymous questionnaires were distributed to all physicians and general practitioners (N = 243, response rate – 78.6%), working at Kaunas primary health care level establishments, in October – December 2003.

**Results:**

15 men (7.9%) and 176 women (92.1%) participated in the research, among which 133 (69.6%) were GPs and 58 (30.4%) physicians. Respondents claimed to have chosen to become doctors, as other professions were of no interest to them. Total job satisfaction of the respondents was 4.74 point (on a 7 point scale). Besides 75.5% of the respondents said they would not recommend their children to choose a PHC level doctor's profession. The survey also showed that the respondents were most satisfied with the level of autonomy they get at work – 5.28, relationship with colleagues – 5.06, and management quality – 5.04, while compensation (2.09), social status (3.36), and workload (3.93) turned to be causing the highest dissatisfaction among the respondents. The strongest correlation (Spearmen's ratio) was observed between total job satisfaction and such factors as the level of autonomy – 0.566, workload – 0.452, and GP's social status – 0.458.

**Conclusion:**

Total job satisfaction of doctors working at primary health care establishments in Lithuania is relatively low, and compensation, social status, and workload are among the key factors that condition PHC doctors' dissatisfaction with their job.

## Background

In 1989, after regaining its independence, Lithuania inherited an extremely centralised health care system that mainly conditioned ineffective health care management and resource usage. The above situation was an outcome of the former Soviet health care policy, as it did not encourage people or the state to treasure or safeguard its citizens' health [1]. The Soviet model of medicine that existed in Lithuania was based exclusively on the exaggerated focus and development of the hospital level, whereas the need to develop the primary health care (PHC) level was practically ignored.

The situation has changed a lot though, and the current Lithuanian health policy states that priority shall be given to the development of primary health care [3,2]. Speaking of the very concept of primary health care, it should be stated that it first was offered in 1978 in Alma- Ata, Kazakhstan. Later on the primary health care concept, policy and strategy were further developed in such documents as "Health to Everybody – 2000" and "Health in the 21^st ^Century" [3]. As regards the health care policy, the above documents are considered of utmost importance in Lithuania as well as other EU countries. During the health care reform, the Lithuanian government came to a decision that the greater part of the health care budget should be allocated to primary health care establishments – consolidation of their basis and improvement of respective personnel qualification, which was expected to lead to a rise in the latter's work efficiency. In 1992, the training programme of general practitioners (GPs) was renewed in Lithuanian higher medical schools, and in 1996 the GP Statute was ratified under Lithuanian Health Norm MN 14:1996. District physicians and paediatricians were retrained as GPs, which requires more thorough competence than being a physician, as GPs are supposed to be capable of consulting, diagnosing and providing primary level help in all area. They have to be competent in internal disease treatment and can send patients to secondary level specialists merely in exceptional cases. The number of general practitioners in Lithuania is increasing, and it amounted to 1,150 at the beginning of 2003 [4].

As stated in the announcements of the National Board of Health, the situation in the Lithuanian health system is changing; nevertheless, it faces a number of problems. 78.6 % (1,146 out of 1,458) of GP graduates in 1994–2001 were retrained from district physicians and paediatricians [5]. Another problematic issue is lack of experience in organising their activities. Furthermore, GPs are often delegated many additional functions, such as accounting, work organisation in their establishments, and co-ordination with other institutions. Considerable instability of the legal basis as well as environment makes a serious negative impact too. Besides a great part of those trained as GPs do not follow their professional career, as they are tempted by considerably higher salaries that are offered by pharmaceutical companies and possibilities of working abroad [5]. This tendency may heighten even more now after Lithuania has joined the European Union, as this offers doctors considerably more opportunities to leave the country. The "loss" of doctors has significant economic consequences as well, as training of qualified general practice physicians is rather costly (a two year internship of one GP costs the state about EUR 8,700); therefore, it is essential to retain qualified specialists in the country.

General medicine practice in health care systems of industrialised countries is considered a remarkably well working system that seeks to assess health needs of individuals and to become familiar with their living conditions, taking into consideration their family and social environment [6]. Nevertheless, recent research shows that "the crisis of general medicine" in the West is being widely discussed [6]. In the contemporary era of specialised medicine, the nature of GP's work is a direct opposite of specialisation: in terms of pathology, it covers all areas; in terms of diagnostics, GPs do not have the required equipment to make a diagnosis; working time is unlimited (from 54.1 to 62.2 hours per week); GPs constantly have to negotiate with patients the medicine to be prescribed and are high dependability on patients (calls to homes are not planned). Furthermore, 30 – 60% of cases in GP's work make psychosomatic problems of patients, which requires more psychological rather than medical knowledge. A GP is assessed not only as a specialist, as the above-mentioned criteria tend to be predominant.

One of the ways to look into the existing PHC situation and possible problems is to study job satisfaction of GPs and physicians, as the attitudes people hold toward their jobs are referred to as job satisfaction and this is one of the most widely studied work-related attitudes. Job satisfaction is closely related to the lower level employee turnover and intentions to quit their jobs [7-9]. Since there is a lack of general practice physicians in Lithuania, it is vital to build conditions that would motivate them to stay and work in the Lithuanian health care system, which may be accomplished through the enhancement of job satisfaction at the primary health care level. Job satisfaction is highly important in building up employee motivation and efficiency, as higher job satisfaction determines better employee performance and higher level of patient satisfaction [9].

The aim of this research is to investigate job satisfaction of physicians and general practitioners in primary health care institutions during the health care reform in Lithuania. In 2003, there were 1,608 GPs and physicians working in Lithuanian PHC establishments [4]. The largest number of primary health care institutions were in Kaunas, the second largest city of Lithuania, (27% of all Lithuanian PHC institutions), and GPs provided services to 42% of the city's population. This research studies GPs and physicians who working in Kaunas.

Research tasks:

1. To determine motives for choosing a PHC doctor's profession.

2. To compare total job satisfaction of Lithuanian PHC physicians' and GPs' as well as their intentions to recommend their children to choose a PHC level doctor's profession.

3. To assess factors which determine job satisfaction of PHC physicians, GPs who chose their specialization at higher school, and GPs who were retrained as GPs later in their career.

### Review of research on job satisfaction in health care institutions

Job satisfaction comprises positive and/or negative attitudes held by individuals in respect to their job [10]. Employees with higher job satisfaction believe that the organisation will continue satisfying them for a long time; they care about the quality of their work, are more committed to the organisation, stay in it longer, are more productive, feel responsible for the working environment, and strive to make it satisfying [6-8]. Job satisfaction reduces employee turnover, absenteeism, and the number of thefts at work, which in turn reduces organisational costs [10]. Job satisfaction is closely related to the nature of work, the quality of management and working environment. Not only does it influence good employee performance, it also maintains good employee health and longevity. As to the most valued aspects of satisfaction, they comprise such things as compensation, promotion opportunities, fringe benefits, bonuses, management, co-workers, working conditions, nature of work, communication, security [11,12].

Bronsky and Cook (1994) interviewed 334 Ohayo University graduate physicians [13]. For the interviews, they used Job Discriminative Index questionnaire, in which satisfaction is assessed according to five sub-scales ("work", "co-workers", "management", "current reward" and "promotion opportunities"). The research showed that job satisfaction is not directly related to practical aspects of physicians.

In 1989 American Physical Therapeutics Association (APTA) carried out a survey of hiring and retention aspects [14]. This study showed that physicians were satisfied with their work. The highest job satisfaction was gained from "autonomy", whereas the smallest was given by "reward".

Okerlund et all (1994) research revealed that Utah physicians were satisfied with their job [15]. Respondents indicated the followings factors as the most important and having the greatest effect on their job satisfaction: "working freedom", "assistance in skills development", and "salary and fringe benefits". The survey also showed that the key factors of doctors' willingness to leave their practice comprised such things as big clerical workload, dissatisfaction with reforms, high patient expectations and big clinical workload. While young doctors willing to leave practice mainly emphasised communication problems and big clinical workload, older doctors were mainly discontent with changes in the health care system and big clerical workload. Differences between genders were insignificant.

Rozier' et al (1998) found out that intrinsic factors, such as ethical practice, improvement of patient health, and perception of satisfaction were more important than big salary and important title [16]. These groups of factors were significantly affected by the gender factor, i. e. how employees combine work and family responsibilities.

Barnes (1998) conducted research of job satisfaction among rehabilitation physicians, occupational therapy specialists, and speech pathologists. Set apart, there are three factors that describe job satisfaction: external context factors and internal context factors, as well as internal content factors [17]. External factors that affect job satisfaction comprise a competitive salary and additional rewards such as release from work due to family matters, flexible work schedule, and child raising support. The stated internal context factors were "less substantial but inherent to work". These could be factors that are controlled by external forces and directly affect internal employee satisfaction, such as adequate working hours and workload, stable working environment, and support of administration. Barnes also pointed out internal content factors that, first of all, are controlled by the professional himself/herself and that may affect employee's competence and feeling of effectiveness in the organisation. The author concludes that internal factors have more influence on employee career satisfaction than external.

Speakman et al (1996) conducted a study of 106 Texas (USA) doctors' job satisfaction [18]. Respondents stated that their work was "a challenge" in a positive sense: it enabled them to use their capabilities and was stimulating. The doctors also pointed out that they were given sufficient autonomy at work and independence in decision-making, and were able to learn and improve their work. The doctors, however, were dissatisfied with the clerical aspect of their work.

Dowell et al (2000) conducted research, the purpose of which was to examine job satisfaction and stress in the work of GPs in New Zealand. Their job satisfaction level was rather high [19]. However, rural doctors were less satisfied with their job than those working in cities. Besides GPs who worked individually were less satisfied with their work than group practice GPs. 177 GPs (46%) stated that work affected their physical health, and 220 (57%) stated that they had been thinking about quitting general practice. The main causes of stress and dissatisfaction were as follows: too much bureaucracy and restrictions, health care reforms, long working hours, and work "by the telephone".

## Methods

### Sample

Research was conducted in October – December 2003. Questionnaires were distributed to all Kaunas PHC physicians and GPs (N = 243) in person. The response rate amounted to 78.6 % (191 questionnaire returned).

### Questionnaire Development

Total job satisfaction of the respondents was measured on a seven-point scale, where 7 stood for highly satisfied and 1 for highly dissatisfied.

Intentions to recommend one's children to choose a PHC level doctor's profession were measured on a seven-point scale, where 7 stood for "strongly agree" and 1 for "strongly disagree".

The level of satisfaction with different job characteristics was assessed using a job satisfaction questionnaire that embraced questions related to the PHC work specifics. Eight sections of questions were included in order to assess satisfaction with different job characteristics: ability utilisation at work, workload, colleagues, compensation, creativity, autonomy, management, and social status. All statements were measured on a seven-point scale, where 7 stood for highly satisfied and 1 for highly dissatisfied.

The survey data was processed using SPSS version 11.00 statistical package for data analysis. The statistical data reliability was checked according to χ^2 ^criteria, degrees of freedom number (df) and statistical significance. Relationship between two independent variables was assessed relying on Spearmen's rank correlation, taking into consideration the value of the correlation ratio and statistical significance. Reliability notation: p < 0.05 – statistically significant, p < 0.01 – highly significant.

## Results

### Demographic characteristics of the respondents

15 men (7.9%) and 176 women (92.1%) participated in the research, among which 133 (69.6%) GPs and 58 (30.4%) physicians.

The analysis of the distribution of the respondents according to their age and specialization (see Table 1) revealed that GP and physician distribution according to their age statistically varied: the percentage of GPs in younger age groups (25–34, 35–44, 45–54 years) is higher than that of physicians. The reason for this is that the specialisation of physicians is no longer included into higher school programmes; only GPs are trained there, and increasingly more young doctors retrain from internists to GPs [5]. Comparing the data of January 1998 and December 2002, we can see that the number of GPs increased by 6.6 times, whereas the number of physicians decreased by 42.6%.

**Table 1:** Characteristics of the respondents

Age group (years)	Specialisation				Total	
			
	GPs*		Physicians*			
	
	n	%	n	%	n	%
25–34	23	17.3	1	1.7	24	12.6
35–44	28	21.1	8	13.8	36	18.8
45–54	73	54.9	16	27.6	89	46.6
55–64	9	6.8	26	44.8	35	18.3
65 and more	-	-	7	12.1	7	3.7
Total	133	100.0	58	100.0	191	100.0

* - χ^2 ^= 63.360; df = 4; p = 0.000

### Motives of choosing phc doctor's profession

With the health care reform underway, it is of great importance to determine how GPs became who they are, and what motives made them chose this specialisation. During the survey, the respondents were divided into 3 groups: the first group comprised GPs who had requalified from other specialisations, the second – GPs who chose this specialisation while studying at higher school, and the third one comprised physicians. In all three groups, doctors admitted to have chosen their profession mostly because no other profession was of interest to them. This motive was indicated by 80% of GPs who chose their specialisation at higher school, 63.4% of GPs who changed their qualification, and 77.4% of physicians. The dominating motive of "no other speciality being of interest" can be explained by the fact that the work of a doctor is easiest to imagine and perceive.

### Total job satisfaction and intentions to recommend one's children to choose a phc level doctor's profession

Total job satisfaction of the respondents was 4.74 point. Recommending a profession to one's own child makes an important factor that shows if an employee is satisfied with his/her work or not. Most respondents (75.5%) do not intend to recommend their children to choose a PHC level doctor's profession. The assessment of the respondents' general satisfaction with work and their intentions to recommend their children to choose a primary level doctor's profession did not reveal any statistically significant differences between the specialisations (p > 0.05). However, the research revealed a tendency among physicians to be generally more satisfied with their work and more willing to recommend their children to choose a PHC level doctor's profession: total job satisfaction of physicians was 5.03 and intentions to recommend their children to follow their career comprised 2.56, while those of GPs respectively made 4.64 and 2.19.

### Satisfaction with different job characteristics

The study of the satisfaction level with different job characteristics shows that respondents were most satisfied with autonomy at work – 5.28, relationship with colleagues – 5.06, and management quality – 5.04 (see Table 2), while compensation (2.09), social status (3.36), and work load (3.93) turned to be causing the highest dissatisfaction among the respondents. Statistically significant differences of satisfaction between the three categories of respondents were determined with two job characteristics, i.e. compensation and social status. GPs who chose specialisation at higher school were more satisfied with compensation (2.68) and social status (4.03) than GPs who changed their qualification (respectively 2.00 and 3.09) and physicians (respectively 1.90 and 3.45) (see Table 2).

**Table 2:** Means score of satisfaction with job characteristics according to specialization and its choice conditions

Job characteristics	Specialisation and its choice			Mean score of satisfaction (standard deviation)
		
	GPs who have requalified (standard deviation)	GPs who chose specialisation at higher school (standard deviation)	Physicians (standard deviation)	
Ability utilisation at work	4.79 (1.50)	4.51 (1.60)	5.18 (1.56)	4.84 (1.54)

Volume of work	3.69 (2.00)	4.29 (1.77)	4.24 (2.11)	3.93 (2.00)
Co-workers	5.05 (1.66)	5.26 (1.75)	4.92 (1.68)	5.06 (1.68)
Compensation*	2.00 (1.49)	2.68 (1.80)	1.90 (1.250)	2.09 (1.51)
Creativity	3.96 (1.66)	4.74 (1.80)	4.30 (1.83)	4.19 (1.75)
Autonomy	5.12 (1.65)	5.23 (1.62)	5.65 (1.25)	5.28 (1.57)
Management	4.86 (1.78)	5.17 (1.74)	5.30 (1.84)	5.04 (1.79)
Social status*	3.09 (1.76)	4.03 (2.01)	3.45 (1.85)	3.36 (1.85)

* – p < 0.05

In order to determine the main factors that cause satisfaction and/or dissatisfaction with work, the relationship between total job satisfaction and job characteristics was analysed. Calculations of Spearmen's ratios revealed the strongest correlation between total job satisfaction and such characteristics as autonomy at work (0.566), workload (0.452) and GP's social status (0.458) (see Table 3). When studying the relationship between the intention to recommend one's children to choose a PHC level doctor's profession and averages of satisfaction with job characteristics, the strongest correlation was again found between the intention to recommend one's children to choose a PHC level doctor's profession and autonomy at work (0.371) as well as GP's social status (0.329).

**Table 3:** Spearmen's ratio between job characteristics and total job satisfaction, and intention to recommend one's children to choose a GP's profession

Job characteristics	Total job satisfaction	Intention to recommend one's own children to choose a GP's profession
Ability utilisation at work	0.402**	0.241**
Volume of work	0.452**	0.228**
Co-workers	0.349**	0.239**
Compensation	0.240**	0.255**
Creativity	0.383**	0.151*
Autonomy	0.566**	0.371**
Management	0.358**	0.175*
Social status	0.458**	0.329**

** – p < 0.01; * – p < 0.05

## Discussions and conclusions

Taking into consideration PHC level doctors' satisfaction with various aspects of work, a conclusion can be drawn that the health care reform that is being implemented meantime and that makes general practice physician the key figure and which should encourage expansion of GP institution is channelled in the right direction. Relying on the data of the conducted research, GPs who chose specialisation while studying at higher school are more satisfied with some aspects of work than physicians and GPs who requalied. However total job satisfaction of doctors who work in the primary health care is relatively low (4.74 on a 7 point scale). Doctors who have worked for 30 – 39 years are most satisfied with their job. Total job satisfaction of GPs and physicians does not have statistically significant difference. Most PHC level doctors (75.5%) do not intend to recommend their children to follow their career.

Such characteristics as autonomy at work, social status, and workload have the biggest impact on total job satisfaction. Stamps and Piedmonte's (2000) research shows that the more autonomy activities possess and the less monotony exists at work, and the more employees are satisfied with their work [20]. The research revealed that compensation, social status, and workload are among the key factors causing PHC doctors' dissatisfaction with their jobs. Thus it can be assumed that the above factors condition low total job satisfaction.

In conclusion it can be stated that the nature of a PHC doctor's work and rather low salaries condition relatively low job satisfaction among Lithuanian PHC doctors.

## Competing interests

The author(s) declare that they have no competing interests.

## Authors' contributions

These authors contributed equally to this work.

## Pre-publication history

The pre-publication history for this paper can be accessed here:


